# Synergistic effect of stromelysin-1 (matrix metalloproteinase-3) promoter (-1171 5A->6A) polymorphism in oral submucous fibrosis and head and neck lesions

**DOI:** 10.1186/1471-2407-10-369

**Published:** 2010-07-14

**Authors:** Ajay K Chaudhary, Mamta Singh, Alok C Bharti, Mangal Singh, Shirish Shukla, Atul K Singh, Ravi Mehrotra

**Affiliations:** 1 Centre for Biotechnology, University of Allahabad, 211002 India; 2 Department of Pathology, Moti Lal Nehru Medical College, Allahabad, 211002 India; 3 Division of Molecular Oncology, Institute of Cytology and Preventive Oncology (ICPO), NOIDA, 201301, India; 4 Department of Otolaryngology, Moti Lal Nehru Medical College, Allahabad, 211002 India; 5 Department of Psychology, Allahabad Degree College, University of Allahabad, 211003 India

## Abstract

**Background:**

Matrix metalloproteinases (MMPs) are enzymes that degrade all the components of extra cellular matrix and collagen. Various types of MMPs are known to be expressed and activated in patients with oral submucous fibrosis (OSMF) as well as head and neck squamous cell carcinoma (HNSCC). The purpose of this study was to asses the association of the single nucleotide polymorphism (SNP) adenosine insertion/deletion polymorphism (-1171 5A->6A) in the MMP-3 promoter region in these lesions.

**Methods:**

MMP-3 SNP was genotyped by polymerase chain reaction-restriction fragment polymorphism (PCR-RFLP) analysis in a case control study consisting of 362 participants; 101 cases of OSMF, 135 of HNSCC and 126 controls, compared for age, sex and habits. ROC distribution was plotted to assess the contributions of genetic variation in MMP-3 genotypes with relation to age.

**Results:**

Analysis of MMP 3 (-1171 5A->6A) polymorphism revealed the frequency of 5A allele in OSMF, HNSCC and controls to be 0.15, 0.13 and 0.07, respectively. A significant difference was found in 5A genotype frequency between OSMF (5A genotype frequency = 0.15, p = 0.01, OR = 2.26, 95% CI = 1.22-4.20) and in controls (5A genotype frequency 0.07) as well as HNSCC (5A genotype frequency 0.13, p = 0.03,95%CI = 1.06-3.51) and controls (5A genotype frequency = 0.07) In this study, 5A genotype had greater than two fold risk for developing OSMF (OR = 2.26) and nearly the same in case of HNSCC (OR = 1.94) as compared to controls. In patients with OSMF as well as HNSCC, the ROC analysis between the MMP-3 genotype and age, 6A/6A allele was found to be significant in patients both over and under 45 years of age; while the 5A/5A carrier alleles showed an association only in patients less than 45 years of age.

**Conclusions:**

This study concluded that the expression of MMP-3 genotype associated with the 5A alleles, it may have an important role in the susceptibility of the patients to develop OSMF and HNSCC.

## Background

Matrix metalloproteinases (MMP) are a group of structurally related zinc-dependent endopeptidases and have the capability to degrade all components of extra cellular matrix (ECM) [1]. MMPs are synthesized mainly by fibroblasts and to a lesser extent by activated macrophages and keratinocytes adjacent to the sites of injury [2]. MMP-3, also known as stromelysin-1, it is responsible for degradation of collagen type IV [3]. Brinckerhoff et al reported that MMP-3 also plays an important role in activation of proMMP-1 into the active form of MMP-1 in malignant tissues [4]. MMP-3 expression is low in normal tissues but it is altered during tumour formation, where remodeling of the extra cellular matrix is required [5]. MMP-3 has wide substrate specificity for various ECM components and is involved in many biological functions, including extracellular matrix degradation and remodelling, cell proliferation, angiogenesis as well as induction of synthesis of other metalloproteinases such as MMP-1 and MMP-9 [6]. The author's group recently reviewed the molecular functions and single nucleotide polymorphic association of different MMPs such as MMP-1 (-1607 1G/2G), MMP-2 (-1306 C/T), MMP-3 (-1171 5A/6A), MMP-9 (-1562 C/T) and TIMP-2 (-418 G/C or C/C) and it's possible therapeutic aspects of these proteases and they concluded that MMPs may play an association with potentially malignant (OSMF) and malignant head and neck lesions (HNSCC). Further research is required for the development of their potential diagnostic and therapeutic possibilities [7].

The expression of MMP-3 in carcinogenesis is regulated primarily at the transcriptional level, but there is also evidence of modulation of mRNA stability in response to growth factors and cytokines secreted by tumour-infiltrating inflammatory cells as well as by tumour and stromal cells [3,5,8]. MMP-3 transcription is higher in head and neck squamous cell carcinoma (HNSCC) and several other types of malignancies including lung and breast carcinomas [9-12]. MMP-3 degrades types II, V, IX and X collagens, proteoglycans, gelatine, fibronectin, laminin and elastin [13]. MMP-3 can also activate other MMPs, including collagenase, matrilysin and gelatinase B.

The MMP-3 gene has been mapped to the long arm of chromosome 11q22.3 and the level of expression of this gene can be influenced by single nucleotide polymorphisms (SNPs) in the promoter region of their respective gene [14]. The promoter region of MMP3 is characterized by a 5A/6A promoter polymorphism at position -1171 in which one allele has six adenosine (6A) and the second has five adenosine (5A). A single adenosine insertion/deletion polymorphism (5A/6A) at position -1171 of the MMP-3 promoter region causes different transcription of MMP-3.

In-vitro assays of promoter activity showed that the 5A allele had a two-fold higher promoter activity than the 6A allele [15]. The 5A allele is more prevalent in the European population (frequency range 40-50%) as compared to East Asian population (frequency range 7-20%) [11,14,16]. At position -1171 of the promoter region of the MMP-3 gene, a polymorphism of 5 or 6 adenosines (5A/6A) affects its low transcription level. The 5A allele results in higher gene expression in fibroblasts and vascular smooth muscle cells compared to the 6A allele and the 6A allele has a lower promoter reactivity than the 5A allele in vitro [14].

The aim of this study was to find out the association of the -1171 5A->6A polymorphism in the MMP-3 gene in patients suffering from OSMF, HNSCC and healthy controls.

## Methods

Patients with OSMF and HNSCC as well as controls from the Departments of Otorhinolaryngology and Pathology, Moti Lal Nehru Medical College, Allahabad, India, were included in the study from June 2007 to October 2009; this study was approved by the institutional ethical committee. This case-control study group included 362 participants. Two hundred and thirty six subjects (average age 43.4 ± 14.8 years) were included in this study, of which 101 patients suffered from OSMF (78 males and 23 females) and 135 patients from HNSCC (114 males and 21 females) and 126 healthy subjects who did not have a history of pre-malignant or malignant lesions. These (average age 37.01 ± 12.89 years, 105 males 21 females) healthy controls were compared for age, sex and habits. Detailed clinical and personal information of each patient was noted in a standardized proforma. Information regarding the patients name, age, sex, occupation, personal habits and present complaints was gathered. Emphasis was given to addictions like areca nut, tobacco and alcohol. None of the HNSCC patients gave a history of having OSMF prior to developing malignancy. Individuals with haematological diseases, previous malignancies, skin diseases and autoimmune disorders were excluded from the study. All cases were histopathologically confirmed. The blood samples were taken after obtaining the patients informed consent to participate in the study. 5 ml. blood was drawn from each subject into vacutainer tubes containing ethylene-di-amine-tetra-acetic acid **(**EDTA) and stored at 4°C till the samples were processed and isolation of genomic DNA was done.

### Isolation of Genomic DNA

Genomic DNA was extracted from the blood samples by using the Qiagen QIAamp DNA Blood Mini Kit (Qiagen Inc. USA) The extracted genomic DNA was quantified and checked for purity by spectrophotometer (Spectro UV-Vis Double Beam PC, UVD Model 2950 LABOMED, Inc. CA, USA). Ethidium bromide (EtBr) stained 0.8% agarose gel electrophoresis was used to confirm the presence of genomic DNA in patient and controls samples.

### Genotyping of the MMP-3 promoter polymorphism

The MMP-3 genotype was determined by the PCR-RFLP assay. The PCR primers used for amplifying the MMP-3 polymorphism were: forward primer (FP) 5'-GGTTCTCCATTCCTTTGATGGGGGGAAAGA-3' and reverse primer (RP) 5'-CTTCCTGGAATTCACATCACTGCCACCACT-3' [17]. An A to G mutation at the second nucleotide close to the 3' end of the FP was made to create a *Tth111I *(TakaRa Biotechnology Co. Ltd, Dalian, China) recognition site in the case of a 5A allele. PCR (MJ Research PTC 100, GMI, Inc, Minnesota, USA) was performed in a 25 μl volume containing 50 ng of genomic DNA template, 2.5 μl of 10× PCR buffer, 2.5 mmol of MgCl_2_, 1 U of Taq DNA polymerase (Fermentas Inc. Glen Burnie MD), 200 nmol of dNTPs and 200 nmol of forward and reverse primer. The PCR cycling conditions were 5 min at 94°C followed by 35 cycles of 45 s at 94°C, 45 s at 66°C and 45 s at 72°C and with a final step at 72°C for 15 min to allow for the complete extension of all PCR fragments. For a negative control, each PCR reaction used distilled water instead of DNA in the reaction mixture.

### Restriction enzyme (Tth 111I) digestion of MMP-3 gene

**Figure 1:**
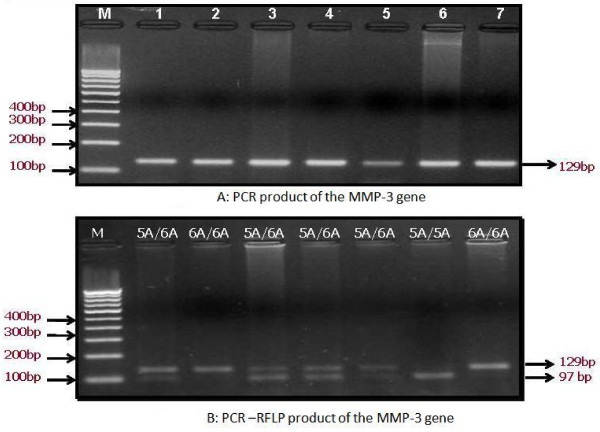
**A The expected PCR product size of MMP-3 gene by 100 bp marker [M], Lane 1-7 shows PCR product followed by separation on 2.0% agarose gel was confirmed**; **B**:** Genotyping of MMP-3 Promoter (-1171 5A->6A) Polymorphism by PCR-RFLP analysis followed by separation on 3. 5% agarose gel, Lane M = 100 bp marker; lane 1, 3, 4, 5 = 5A/6A; lane 2 = 5A/6A; lane 6 = 5A/5A.**

A 10 μl aliquot of PCR product was digested at 65°C for 5 hrs in a 15 μl reaction containing 10 U of *Tth111 I *and 1× reaction buffer. After digestion, the products were separated on 3.5% agarose gel stained with EtBr. On electrophoresis, the 5A alleles were represented by DNA bands of 97 and 32 bp, the 6A alleles were represented by a DNA band of 129 bp, whereas the heterozygote displayed a combination of both alleles (129, 97 and 32 bp) Fig. 1[A] and 1[B].

### Statistical analysis

The Chi-square test **(**χ^2 ^test) was used to find out the difference in genotypic distribution of MMP-3 promoters between the OSMF, HNSCC and control groups. Fisher's exact test has also been applied in the statistical analysis. A p-value of < 0.05 was considered as statistically significant. For each parameter, patients with OSMF histopathological grade I, II, III, IV and HNSCC, risk was analyzed by odds ratios (OR) and 95% Confidence Intervals (95% CI). Distributions of MMP-3 genotype promoter polymorphisms in OSMF, HNSCC and control groups were analyzed by the Hardy-Weinberg equilibrium. The statistical analysis was performed using the SPSS 15.0 software package (SPSS Japan Inc., Tokyo, Japan).

## Results

**Figure 2:**
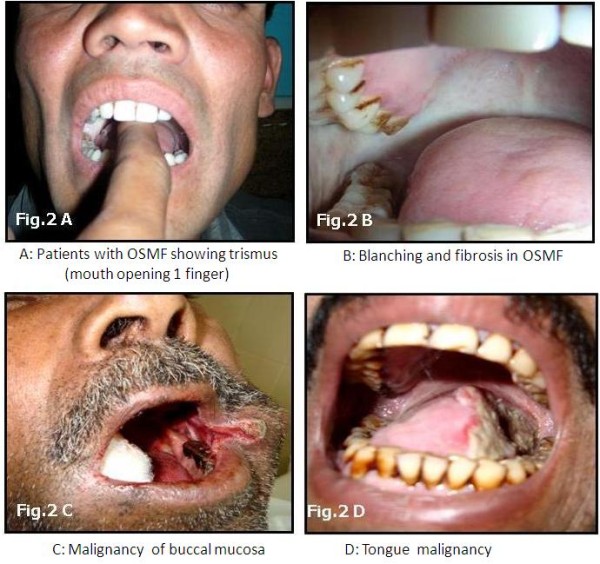
**Clinical picture of patients with OSMF and HNSCC**.

Patients of OSMF were diagnosed on the basis of clinical signs including trismus and presence of fibrotic bands in the oral cavity and malignancies. Figure 2[A, B, C] and 2[D]. MMP-3 promoter genotypes in patients with OSMF, HNSCC and controls were analyzed with respect to gender, age, habits like tobacco consumption with or without areca nut chewing, alcohol intake, as well as histopathological grade of OSMF, TNM staging of HNSCC and the location of the lesion.

Out of 101 patients with OSMF, 74 (73.27%) had 6A/6A genotype, 24 (23.76%) 5A/6A and 3 (2.97%) 5A/5A, while in 135 patients with HNSCC, 106 (78.5%) had 6A/6A genotype, 23 (17.03%) 5A/6A and 6 (4.44%) 5A/5A. Of the male patients with OSMF, 62 had 6A/6A, 14 (5A/6A) and 2 (5A/5A) genotype and of the female patients, 12 had 6A/6A, 10 (5A/6A) and 1 (5A/5A) genotype. In male patients with HNSCC, 94 had 6A/6A, 15 (5A/6A) and 5 (5A/5A) genotype and of the females, 12 had 6A/6A, 8 (5A/6A) and 1 (5A/5A) genotype. Analyzing the different genotypes (6A/6A, 5A/6A and 5A/5A), it was found that the polymorphic association of these genotypes was statistically significant in both male and females suffering from OSMF (p = 0.001) and HNSCC (p = 0.01), according to the Fisher's exact test. This conclusion was drawn on analysis of the dataset illustrated in Table no 1.

Association of 5A/5A, 6A/6A and 5A/6A genotypes and tobacco chewing with areca nut chewers showed a significant association in OSMF and HNSCC cases respectively (p = 0.02,0.003) as did the OSMF (p = 0.001) and HNSCC histopathological grading (p = 0.001) as well as location, both in OSMF and HNSCC (p = 0.81 and p = 0.59 respectively) [Table 1].

**Table 1:** Relation of MMP-3 genotype to clinical parameters and environmental factors among the OSMF, HNSCC and Control.

Characteristics		OSMF (N = 101)				HNSCC (N = 135)				Total Control	Control (N = 126)			
				
		6A/6A genotype	5A/6A genotype	5A/5A genotype	p-value	6A/6A genotype	5A/6A genotype	5A/5A genotype	p-value		5A/5A genotype	6A/6A genotype	5A/6A genotype	(p-value)
		
Total	N = 236	74 (73.27%)	24 (23.76%)	3 (2.97%)		106 (78.5%)	23 (17.03%)	6 (4.44%)		126	2 (1.6%)	110 (87.3%)	14 (11.1%)	
**Gender**														
Male	192 (81.35)	62(83.8)	14(58.3)	2(66.6)	0.001	94 (88.67)	15(65.21)	5 (83.3)	0.01	105(83.3)	2 (100)	101 (91.8)	2(14.3)	0.01
Female	44 (18.64)	12(16.2)	10 (41.6)	1(33.3)		12 (11.32)	8(34.78)	1 (16.6)		21(16.7)	0	9(8.2)	12(85.7)	

**Age (Yrs)**														
Mean ± SD	43.4 ± 14.8	38.8 ± 14.5	37.6 ± 14.2	36 ± 8.5	0.671	42 ± 16.5	47 ± 10.8	41 ± 9.86	0.42	37.0 ± 12.8	17.5 ± 0.7	36.7 ± 12.4	38.8 ± 16	0.41

**Tobacco with Areca nut**														
Chewer	215(91.1)	72(97.3)	23 (95.8)	2 (66.7)	0.02	98(92.45)	16(69.56)	4 (66.6)	0.003	85(67.5)	2(100)	71(64.5)	12(85.7)	0.12
Non Chewer	21(8.9)	2(2.7)	1 (4.2)	1 (33.3)		8(7.54)	7(30.43)	2 (33.3)		41(32.5)	0	39(35.5)	2(14.3)	

**Smoking status**														
Smoker	170(72.03)	42(56.7)	19(79.2)	3 (100)	0.077	88(83.01)	15(65.21)	4 (66.6)	0.037	51(40.5)	0	47(42.7)	4(28.6)	0.11
Non-smoker	66 (27.9)	32(43.3)	5 (20.8)	0		18(16.98)	8(34.78)	2 (33.3)		75(59.5)	2(100)	63(57.3)	10(71.4)	

**Alcohol Intake**														
Drinker	117(49.57)	22(29.7)	3 (12.5)	2 (66.7)	1.16	72(67.92)	14(60.86)	3 (50)	0..81	26(20.6)	0	25(22.7)	1(7.1)	0.24
Non Drinker	119(50.42)	52(70.3)	21 (87.5)	1 (33.3)		34(32.07)	9(39.1)	3 (50)		100(79.4)	2(100)	85(77.3)	13(92.9)	

**Location**														
Lip (lower &upper)	51 (21.61)	20(27)	7 (29.2)	1 (33.3)		19(17.92)	3(13.04)	1(16.67)						
Tongue	38 (16.1)	10(13.5)	4 (16.7)	0		21(19.81)	2(8.69)	1 (16.1)						
Right & left Cheek	32(13.56)	11(15)	3 (12.5)	1 (33.3)	0.81*	14(13.20)	3(13.04)	1 (16.1)	0.59*	-	-	**-**	**-**	**-**
Buccal mucosa	34 (14.41)	10(13.5)	4 (16.6)	0		15(14.15)	3(13.04)	1 (16.1)						
Larynx/pharynx	81 (34.32)	23(31)	6(25)	1 (33.3)		37(34.91)	12(52.17)	2 (33.3)						

**OSMF Grade**														
**(N = 101)**														
I-II	73 (72.28)	55 (74.3)	16 (66.6)	2 (66.6)	0.001	-	-	-		-	-	**-**	**-**	**-**
III-IV	28 (27.72)	19 (25.7)	8 (33.3)	1 (33.3)										
**HNSCC Grade**														
**(N = 135)**														
**T category**														
T1-3	37 (27.41)					21 (19.81)	14 (60.86)	2 (33.3)						
T4	98 (72.59)	-	-	-	-	85 (80.18)	9 (39.13)	4 (66.6)	0.001					
**N category**														
N0														
N1-3	42 (31.11) 93 (68.89)	-	-	-	-	30 (28.31) 76 (71.69)	6 (26.1) 17 (73.9)	1 (16.7) 5 (83.3)	0.814					

T & N categories [UICC TNM classification], Fisher's exact test & * Pearson's χ^2 ^test were applied for statistical analysis.

The MMP-3 genotype distinguished homozygous 6A genotype (6A/6A), homozygous 5A genotype (5A/5A) and heterozygous genotype (5A/6A). The 5A allele frequencies in OSMF, HNSCC and controls were 0.15, 0.13 and 0.07 respectively (According to Hardy-Weinberg equilibrium). A significant difference was found in 5A allele frequency between OSMF and controls (p = 0.01), as well as HNSCC and controls (p = 0.03). 5A genotype had a more than two fold risk for OSMF (OR = 2.26) and little less in HNSCC (OR = 1.94) in relation to the control group. Frequency of 5A/6A or 5A/5A promoter genotypes having 5A alleles was associated with MMP-3 single nucleotide polymorphism (SNP) in OSMF (5A allele frequency = 0.15, p = 0.01, OR = 2.26, 95% CI = 1.22-4.20). Similar difference was also found in MMP-3 genotype distribution in HNSCC (5A allele frequency = 0.13, p = 0.03, OR = 1.94, 95% CI 1.06-3.51) as compared to controls (5A allele frequency = 0.07) [Table 2].

**Table 2:** The genotype and allele frequency of MMP-3 in OSMF, HNSCC patients and controls.

Groups	Total (N)	5A/5A Genotype	5A/6A Genotype	6A/6A Genotype	5A allele frequency	OR	95%CI	P-value
**Control**	126	2 (1.58%)	14 (11.1%)	110 (87.3%)	0.07	1	-	-

**OSMF**	101	3(2.9%)	24 (23.7%)	74 (73.3%)	0.15	2.26	1.22-4.20	0.01

**HNSCC**	135	6 (4.44%)	23 (17.03%)	106 (78.52%)	0.13	1.94	1.06-3.51	0.03

[OR = Odd Ratio; CI = confidence interval; MMP = matrix metalloproteinase; OSMF = oral submucous fibrosis; HNSCC = Head and neck squamous cell carcinoma]

**Figure 3:**
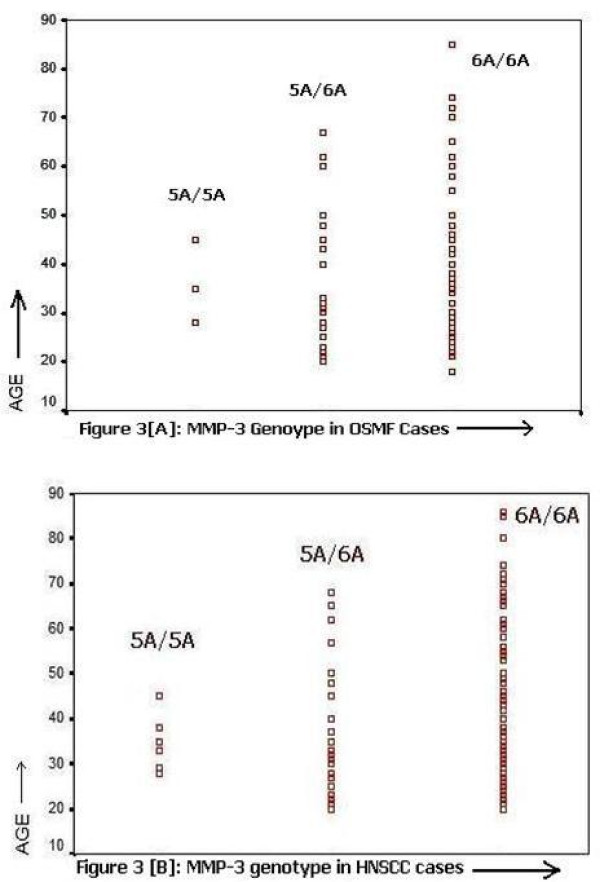
**ROC 6A/6A allele was also found to be significant in subjects both over and under 45 years of age, while 5A/5A carrier alleles showed an association only in patients less than 45 years of age in case of [A] OSMF and [B] HNSCC**.

Receiver operating characteristics (ROC) showed a significant association between age and MMP-3 genotypes in OSMF and HNSCC. Figure 3[A] and 3[B] shows that 6A/6A allele was also found to be significant in subjects both over and under 45 years of age. While, 5A/5A carrier alleles showed an association only in patients less than 45 years of age. Fisher's exact test also revealed that patients with 5A alleles had a significant risk to develop OSMF (p = 0.01, OR = 2.51, 95%CI = 1.27-4.94). [Table 3 and 4]. On the other hand, significant difference was found in MMP-3 genotype distribution (6A/6A, 5A/5A and 5A/6A) between the areca nut with tobacco chewers and the non chewers both in OSMF (p = 0.02) and HNSCC (p = 0.003) patients vis-à-vis control group (p = 0.12) [Table 5 and 6]. However it should be noted that there are differences in age distribution, smoking and areca nut chewing between controls and patients.

**Table 3:** Univariate analysis of predictive factors in case of OSMF.

	OSMF	CONTROL	p-value	OR	95%CI
**Age**					
<45	76	64	0.001	2.94	1.66-5.21
≥45	25	62			

**Gender**					
Male	77	105	0.24	0.64	0.33-1.23
Female	24	21			

6A*					
-ve	3	2	0.79	1.89	0.05-6.93
+ve	98	124			

5A**					
-ve	74	110	0.01	2.508	1.27-4.94
+ve	27	16			

*6A-denotes 5A/5A genotypes and 6A+denotes 6A/6A or 5A/6A. **: 5A-denotes 6A/6A genotype and 5A+ denotes 5A/5A or 5A/6A. Data were analyzed by Fisher's exact test.

**Table 4:** Univariate analysis of predictive factors in case of HNSCC.

	HNSCC	CONTROL	p-value	OR	95%CI
**Age**					
<45	16	78	0.001	2.74	0.13-0.54
≥45	36	48			

**Gender**					
Male	114	105	0.93	1.08	0.56-2.10
Female	21	21			

6A*					
-ve	6	2	0.03	2.93	0.58.79
+ve	129	126			

5A**					
-ve	106	110	0.02	1.8	0.96-3.66
+ve	29	16			

* 6A-denotes 5A/5A genotypes and 6A+denotes 6A/6A or 5A/6A. **: 5A-denotes 6A/6A genotype and 5A+ denotes 5A/5A or 5A/6A. Data were analyzed by Fisher's exact test.

**Table 5:** Age and habits distribution in relation to alleles in OSMF and Controls.

Characteristics	OSMF (N = 101)				Control (N = 126)			
	
	6A/6A	5A/6A	5A/5A	p-value	5A/5A	6A/6A	5A/6A	(p-value)
**Age (Yrs)**								
Mean ± SD	38.8 ± 14.5	37.6 ± 14.2	36 ± 8.5	0.671	17.5 ± 0.7	36.7 ± 12.4	38.8 ± 16	0.41

**Tobacco with Areca nut**								
Chewer	72(97.3%)	23 (95.8%)	2 (66.7%)	0.02	2(100%)	71(64.5%)	12(85.7%)	0.12
Non Chewer	2(2.7%)	1 (4.2%)	1 (33.3%)		0	39(35.5%)	2(14.3%)	

**Smoking status**								
Smoker	42(56.7%)	19(79.2%)	3 (100%)	0.077	0	47(42.7%)	4(28.6%)	0.11
Non-smoker	32(43.3%)	5 (20.8%)	0		2(100%)	63(57.3%)	10(71.4%)	

**Alcohol Intake**								
Drinker	22(29.7%)	3 (12.5%)	2 (66.7%)	1.16	0	25(22.7%)	1(7.1%)	0.24
Non Drinker	52(70.3%)	21 (87.5%)	1 (33.3%)		2(100%)	85(77.3%)	13(92.9%)	

[Data was expressed as mean ± SD and %. Fischer exact test is used for habits in cases and control groups]

**Table 6:** Age and habits distribution in relation to alleles in HNSCC and Controls

Characteristics	HNSCC (N = 135)				Control (N = 126)			
	
	6A/6A	5A/6A	5A/5A	p-value	5A/5A	6A/6A	5A/6A	(p-value)
**Age (Yrs)**								
Mean ± SD	42 ± 16.5	47 ± 10.8	41 ± 9.86	0.42	17.5 ± 0.7	36.7 ± 12.4	38.8 ± 16	0.41

**Tobacco with Areca nut**								
Chewer	98(92.45%)	16(69.56%)	4 (66.6%)		2(100%)	71(64.5%)	12(85.7%)	0.12
Non Chewer	8(7.54%)	7(30.43%)	2 (33.3%)	0.003	0	39(35.5%)	2(14.3%)	

**Smoking status**								
Smoker	88(83.01%)	15(65.21%)	4 (66.6%)		0	47(42.7%)	4(28.6%)	0.11
Non-smoker	18(16.98%)	8(34.78%)	2 (33.3%)	0.037	2(100%)	63(57.3%)	10(71.4%)	

**Alcohol Intake**								
Drinker	72(67.92%)	14(60.86%)	3 (50%)	0..81	0	25(22.7%)	1(7.1%)	0.24
Non Drinker	34(32.07%)	9(39.1%)	3 (50%)		2(100%)	85(77.3%)	13(92.9%)	

[Data was expressed as mean ± SD and %. Fischer exact test is used for habits in HNSCC cases and control groups]

## Discussion

Carcinogenesis is a multistage process, which involves various molecular mechanisms associated to the fundamental alterations in cell physiology such as growth signals, escape from apoptosis, uncontrolled replication and growth-inhibitory signals [18]. MMPs are involved in the degradation of extra cellular matrix (ECM) components that take part in several molecular mechanisms such as invasion, angiogenesis, metastasis and progression of malignancies. Nagata et al suggested that expression levels of that molecules which are involved in tissue remodeling and cell-ECM adhesion, especially MMP-1 and integrin-3, may provide an accurate biomarker diagnosis for predicting the risk of cervical lymph node metastasis in oral squamous cell carcinoma (OSCC) [19]. Lin et al reported that functional promoter polymorphisms of MMP-1 and MMP-2 gene might be associated with the risk for development of OSCC but not in OSMF [20,21].

The MMP-3 promoter polymorphism is associated with a higher MMP-3 transcription level, Hashimoto et al reported that the MMP-3 SNP was not associated with the progression of OSCC as reflected by TNM and advances in stage [9]. In this study, a higher frequency of 5A allele was found in OSMF and HNSCC as compared to controls. This study supports the hypothesis that adenine insertion/deletion polymorphism in the MMP-3 promoter region was associated with the increased chance of development of OSMF. Tu et al also earlier reported the similar findings in OSMF patients from Taiwan [22].

MMPs play an important role in fibrosis of the oral cavity. OSMF is a premalignant disorder, which can be induced by areca nut chewing [23]. Due to excess chewing of areca nut; arecoline may increase the production of collagen fibers in the sub epidermal layer of buccal cavity and causes fibrosis. Chiu et al reported that areca chewing as well as polymorphism of genes associated with collagen homeostasis was correlated with risk of OSMF [24]. Shin et al suggested that polymorphism of transforming growth factor (TGF-α) related to an innate immune response was also found to associated with the risk of OSMF [25]. In this study, a significant difference was found in MMP-3 genotype polymorphism between controls, OSMF and HNSCC patients.

Areca nut chewers having a 5A allele had a higher risk for OSMF and HNSCC (OR = 2.26 and 1.94 respectively) in relation to the control group. Tu et al reported a significant difference in MMP-3 genotypic polymorphism between patients with OSMF vis-à-vis controls in male areca chewers, however not in patients with OSCC from Taiwan [22]. On the other hand, Vairaktaris et al from Greece also reported a significant difference in the 5A allele in oral cancer patients who smoked [26].

The presence of the 5A allele has been reported to be associated with susceptibility for oral cancer, lung carcinoma, esophageal squamous cell carcinoma, head and neck carcinoma, ovarian cancer as well as breast carcinomas [22,27-31]. On the other hand, a patient with lower expression of 6A allele has been associated with increased risk for colorectal cancer [32]. Nishijima et al reported that the anti-MMP-3 antibody is a serological marker that reflects the severity of systemic sclerosis and also suggested that it may contribute to the development of fibrosis by inhibiting MMP-3 activity and reducing the ECM [33]. Murawaki et al 1999 reported that measurement of serum MMP-3 is of little use for assessing fibrolysis in chronically diseased livers [34].

Tissue inhibitors of metalloproteinases (TIMPs) are the main endogenous inhibitors of the MMPs [35]. Strup-Perrot et al reported that expression of collagens; MMPs and TIMPs were increased simultaneously due to effect of collagen deposition in abdominal and pelvic cancers in radiation enteritis [36]. Chang et al found that arecoline acted not only as an inhibitor of gelatinolytic activity of MMP-2, but also a stimulator for TIMP-1 activity in OSMF [37]. Their study concluded that MMPs transcription activity might be associated with genesis of OSMF in younger areca nut chewers.

In this study, 6A/6A alleles was found to be significant both in OSMF and HNSCC patients less than and more than 45 years of age, while 5A/5A carrier alleles showed an association in OSMF and HNSCC patients less than 45 years of age. Nishizawa et al reported that apparent reduction of the MMP-1 1G/1G and 1G/2G genotypes distribution among the early onset OSCC cases under the ages of 45 years [38]. Since there were differences in age distribution, smoking and areca chewing between control and patients, the result need to be cautiously interpreted.

McCawley et al reported that high MMP-3 expression levels in OSCC may play an important role in oncogenesis and especially in invasion [39] Vairaktaris et al reported that the genotype containing 5A allele had a two-fold risk of oral cancer development in smokers [26]. In this study, a significant difference was found in MMP-3 genotypic polymorphism between controls and HNSCC (OR = 1.94), which is in concordance with results of Varkataris et al [26].

## Conclusions

We conclude that adenosine insertion/deletion polymorphism (-1171 5A->6A) in the MMP-3 promoter region may play an important role in the development, initiation and progression of these lesions. To the best of our knowledge, this is the first study dealing with MMP-3 polymorphism in OSMF and HNSCC patients of Indian origin. Studies with a larger number of subjects are recommended to elucidate the genetic and polymorphic contribution of MMPs in these lesions, as this may have important role in designing future therapeutic strategies.

## Abbreviations

(MMP): Matrix metalloproteinase; (ECM): Extra cellular Matrix; (SNP): single nucleotide polymorphism; (PCR): Polymerase chain reaction; (RFLP): Restricion fragment length polymorphism; (OSMF): Oral submucous fibrosis; (HNSCC): head and neck squamous cell carcinoma; (OR): odds ratio; (95% CI): 95% Confidence Intervals; (ROC): receiver operating characteristic curve.

## Competing interests

The authors declare that they have no competing interests.

## Authors' contributions

AKC carried out the experimental work, analysis and drafted the manuscript. RM and Mamta Singh conceived of the study, participated in its design and coordination as well as helped to draft the manuscript. MS, ACB and SS participated in coordination of the study and helped to draft the manuscript. AKS participated in the statistical analysis. All authors read and approved the final manuscript.

## Pre-publication history

The pre-publication history for this paper can be accessed here:

http://www.biomedcentral.com/1471-2407/10/369/prepub
